# Supplementary material for: Network-Based Prediction of Side Effects of Repurposed Antihypertensive Sartans against COVID-19 via Proteome and Drug-Target Interactomes
Source: Proteomes. 2023 Jun 8;11(2):21. doi: 10.3390/proteomes11020021 (PMC10305495; doi:10.3390/proteomes11020021)
Supplement: Supplementary file 1 [file proteomes-11-00021-s001.zip › Supplementary_material.pdf]

Supplementary material

Network-Based Prediction of Side Effects of Repurposed Antihypertensive Sartans against COVID-19 via Proteome and Drug-Target Interactomes

Despoina P. Kiouri [1,2,†], Charalampos Ntallis [1,†], Konstantinos Kelaidonis [3], Massimiliano Peana [4], Sotirios Tsiodras [5], Thomas Mavromoustakos [2], Alessandro Giuliani [6], Harry Ridgway [7,8], Graham J. Moore [9,10], John M. Matsoukas [3,10,11,12,*], and Christos T. Chasapis [1,*]

1 Institute of Chemical Biology, National Hellenic Research Foundation, 11635 Athens, Greece; despoina.kiouri.99@gmail.com (D.P.K.); xntallis@gmail.com (C.N.)
2 Department of Chemistry, Laboratory of Organic Chemistry, National Kapodistrian University of Athens, 15772 Athens, Greece; tmavrom@chem.uoa.gr
3 NewDrug PC, Patras Science Park, 26504 Patras, Greece; k.kelaidonis@gmail.com
4 Department of Chemical, Physical, Mathematical and Natural Sciences, University of Sassari, Via Vienna 2, 07100 Sassari, Italy; peana@uniss.it
5 4th Department of Internal Medicine, School of Medicine, National and Kapodistrian University of Athens, 11527 Athens, Greece; sotirios.tsiodras@gmail.com
6 Environment and Health Department, Istituto Superiore di Sanità, 00161 Rome, Italy; alessandro.giuliani@iss.it
7 Institute for Sustainable Industries and Liveable Cities, Victoria University, Melbourne, VIC 8001, Australia; ridgway@vtc.net
8 AquaMem Consultants, Rodeo, NM 88056, USA
9 Pepmetics Inc., 772 Murphy Place, Victoria, BC V6Y 3H4, Canada; mooregj@shaw.ca
10 Department of Physiology and Pharmacology, Cumming School of Medicine, University of Calgary, Calgary, AB T2N 1N4, Canada
11 Institute for Health and Sport, Victoria University, Melbourne, VIC 3030, Australia
12 Department of Chemistry, University of Patras, 26504 Patras, Greece
* Correspondence: imats1953@gmail.com (J.M.M.); cchasapis@eie.gr (C.T.C.)
† These authors contributed equally to this work.

Supplementary excel files table of contents:

1. **Allelic_variants_Protein_Targets_OMIM**

Sheet1: AGTR1_allelic_variants

Sheet2: PPARG_allelic_variants

2. **Alternative_transcription_variants**

Sheet1: AGTR1

Sheet2: PPARG

Sheet3: ACE2

Sheet4: NR1I2

3. **Gene_Disease_First_Neighbors_Sartans_Paxlovid**

Sheet1: AGTR1_JUN_PPARG

Sheet2: AGTR1

Sheet3: NR1I2

Sheet4 ACE2

4. **Gene_Disease_Targets_Sartans_Paxlovid**

Sheet1: AGTR1

Sheet2: JUN

Sheet3: PPARG

Sheet4: NR1I2

Sheet5: ACE2

5. **Drug_Targets_ALL_Drugs_DrugBank**

Sheet1: Sartans

Sheet2: Paxlovid

6. **Post_Translational_Modifications_Uniprot………**

Sheet1: Targets

Sheet2: ACE2_ALL_FN

Sheet3: ACE2_Top10ConnectedFN

Sheet4: AGTR1_ALL_FN

Sheet5: AGTR1_Top10ConnectedFN

Sheet6: NR1I2_ALL_FN

Sheet7: NR1I2_Top10ConnectedFN

7. **Gene_Ontology_BiNGO**

Sheet1: Targets

8. Gene_Ontology_Panther

Sheet1: Sartans_First_Neighbors

Sheet2: Paxlovid_First_Neighbors

Sheet3: AGTR1_First_Neighbors

Sheet4: ACE2_First_Neighbors

9. ALL_Network_Analysis

Sheet1: Sartans_network

Sheet2: Irbesartan_network

Sheet3: Telmisartan_network

Sheet4: Sartans_COVID

Sheet5: Ritonavir (Paxlovid)

10. Structural_simillarity_Tanimoto

Sheet1: Ritonavir

Sheet2: Nirmatrelvir
